# Rapid, sensitive, and highly specific detection of monkeypox virus by CRISPR-based diagnostic platform

**DOI:** 10.3389/fpubh.2023.1137968

**Published:** 2023-06-27

**Authors:** Lin Gong, Xiaomin Chen, Yimei Wang, Jiansheng Liang, Xiaoli Liu, Yi Wang

**Affiliations:** ^1^Department of Disinfection and Pest Control, Wuhan Center for Disease Control and Prevention, Wuhan, Hubei, China; ^2^Experimental Research Center, Capital Institute of Pediatrics, Beijing, China

**Keywords:** monkeypox, monkeypox virus, RPA, CRISPR/Cas12a, MPXV-RCC

## Abstract

**Background:**

Monkeypox (MPX), caused by the Monkeypox virus (MPXV), has incurred global attention since it broke out in many countries in recent times, which highlights the need for rapid and reliable diagnosis of MPXV infection.

**Methods:**

We combined recombinase polymerase amplification (RPA) with CRISPR/Cas12a-based detection to devise a diagnostic test for detection of MPXV and differentiation of its two clades [Central Africa clade (MPXV-CA) and West Africa clade (MPXV-WA)], and called it MPXV-RCC. The sensitivity, specificity and practicability of this method have been analyzed.

**Results:**

The optimal conditions of MPXV-RCC assay include two RPA reactions at 38°C for 25 min and a CRISPR/Cas12a-gRNA detection at 37°C for 10 min. The results of MPXV-RCC assay were indicated by a real-time fluorescence analysis software. Thus, the whole detection process, including rapid template preparation (20 min), RPA reaction (25 min) and CRISPR-based detection (10 min), could be finished within 1 hour. The sensitivity of MPXV-RCC for MPXV-CA and MPXV-WA detection was down to 5~10 copies of recombination plasmids and pseudovirus per reaction. Particularly, MPXV-RCC assay could clearly differentiate MPXV-CA from MPXV-WA, and had no cross-reactivity with other pathogens. In addition, the feasibility of MPXV-RCC assay was further validated by using spiked clinical samples.

**Conclusion:**

The MPXV-RCC assay developed here is a promising tool for quick and reliable diagnosis of MPXV infection.

## Introduction

As the ongoing COVID-19 pandemic is still challenging the world, a new threat to public health caused by a global surge of monkeypox (MPX) cases has emerged (1). MPX, as a re-emerging zoonotic disease, is caused by the monkeypox virus (MPXV), which is a double-stranded DNA virus with typical virus sizes varying from 200 to 250 nm (2). MPXV is a member of the *orthopoxvirus* genus in the *poxviridae* family and has two genetic clades: Central Africa (MPXV-CA) and West Africa (MPXV-WA). Particularly, MPXV-CA strains exhibited more virulent effects (2). Monkeypox could spread among populations through close contact *via* respiratory droplets, body fluids, and lesions of infected people or animals (3). Until now this year, a total of more than 80,000 MPX cases have been reported in 110 member states of the WHO (4). The primary affected countries included the United States of America, Spain, Germany, the United Kingdom, and France (4). Hence, a rapid and reliable tool for MPXV detection will be helpful to control the dissemination of this virus.

Although antigen and serology-based methods have been used for the diagnosis of MPXV in the early days (5), they have cross-reaction with other *orthopoxviruses* (6). Currently, the definitive confirmation of MPXV infection relies on polymerase chain reaction (PCR) and real-time PCR according to WHO guidelines (6, 7). However, these PCR-based methods are time-consuming and strongly rely on skilled personnel, complex instruments, and a stable power supply. Hence, further development of easy-to-use, simple, and more rapid techniques to diagnose MPXV infection is still needed.

Clustered regularly interspaced short palindromic repeat (CRISPR) and CRISPR-associated protein (Cas) techniques known as “molecular scissors” are used to edit the genome for its efficient and accurate capacity (8). Particularly, the CRISPR/Cas system has been applied as an appropriate tool for nucleic acid detection due to its sensitivity and specificity (9). Various Cas enzymes, such as Cas12a, Cas13, and Cas14, have been developed successfully and show an excellent prospect in nucleic acid diagnosis (10–12). Cas12a, Cas13, and Cas14 enzymes target double-stranded DNA (dsDNA), single-stranded RNA (ssRNA), and single-stranded DNA (ssDNA), respectively (8). Under the direction of guide RNA (gRNA), the aforementioned enzymes could specifically recognize and cleave the targeted nucleic acid sequence. Simultaneously, the non-specific cut capacity of the Cas enzymes is activated to cleave the surrounding ssDNA (Cas12a and Cas14) and ssRNA (Cas13) (8, 13). In this connection, ssDNA and ssRNA could be prepared into fluorescent probes; thus, the produced fluorescent signal will be detected by molecular sensors when cleavage occurs (14).

Owing to omitting sophisticated equipment and saving time with isothermal amplification techniques, the CRISPR/Cas system usually couples with them on the pre-augmented phase to improve the detection sensitivity (14). Until now, several CRISPR-based methods have been implemented in microbe surveillance. For example, multiple cross displacement amplification with CRISPR/Cas12a-based detection (MCCD) was developed for the diagnosis of SARS-CoV-2 (13); the CRISPR/Cas12a-based RPA method was devised to detect severe fever with thrombocytopenia syndrome virus (SFTSV), influenza A virus, and *Mycoplasma hominis* (15–17); similarly, CRISPR/Cas13a coupled with the loop-mediated isothermal amplification (LAMP) assay was contrived for monitoring circular RNA and SARS-CoV-2 (18, 19).

In this study, we coupled recombinase polymerase amplification (**R**PA) with **C**RISPR/**C**as12a-based detection to establish a sensitive and specific diagnostic test for rapid detection of MPXV and accurate differentiation of MPXV-CA and MPXV-WA strains and named it MPXV-RCC. The whole detection procedure could be finished within 60 min, which is thus suitable for point-of-care detection. We illustrated the basic principle and operational process of the MPXV-RCC assay and verified the feasibility by using spiked specimens.

## Materials and methods

### Target DNA and clinical specimens

In this study, two kinds of plasmids (MPXV-CA recombination plasmid and MPXV-WA recombination plasmid) and MPXV pseudovirus were constructed and used as positive controls. The construction of plasmids and pseudovirus has been expounded as follows. The gene sequence of the MPXV D14L gene (651 bp in length, GenBank KJ642613.1) was synthesized and cloned into *E. coli* pUC57 vector (2,710 bp in length) to specifically construct an MPXV-CA recombination plasmid (3 ng/μl), and the copies of plasmid were calculated using a formula: copies/μl = concentration(ng/μl) × 10^−9^ × 6.02 × 10^23^ (copies/mol)/(sequence length × 660). Thus, the concentration of the MPXV-CA recombination plasmid was 8.1 × 10^8^ copies/μl according to the calculation. Similarly, the MPXV-WA recombination plasmid (8.8 × 10^8^ copies/μl) was synthesized by inserting the specific sequence of the ATI gene (392 bp in length, GenBank DQ011156.1) into *E. coli* pUC57 vector as well. Two plasmids were constructed by Tianyi-Huiyuan Biotechnology (Beijing, China), and they acted as a positive template to explore the optimal reaction condition of the MPXV-RCC assay. MPXV pseudovirus was purchased from Sangon Biotechnology (Shanghai, China). MPXV partial sequences containing both the D14L and ATI genes were cloned *in vitro* and constructed into adenovirus vector Ad5, which was used to induce pseudovirus in 293A cells. After purification by chromatography column, the pseudovirus was obtained in the form of a DNA sequence encapsulated by an adenovirus capsid. Pseudovirus concentration (1.0 × 10^5^ copies/μl) was quantified according to the standard curve of qPCR.

In addition, a total of 35 nucleic acid samples extracted from non-MPXV strains were employed in this study (Supplementary Table S1), and each microorganism was verified using real-time PCR. A recombinase-mediated isothermal amplification kit for RPA reaction was obtained from HuiDeXin Biotechnology Development (Tianjin, China). A LbaCas12a protein kit applied to CRISPR/Cas12a cleavage was purchased from Magigen Biotechnology (Guangzhou, China). The emitted fluorescence signals could be captured by a real-time PCR thermocycler instrument (LightCycler 480, Roche, Basel, Switzerland).

### Primers and CRISPR gRNA design

**Figure 1:**
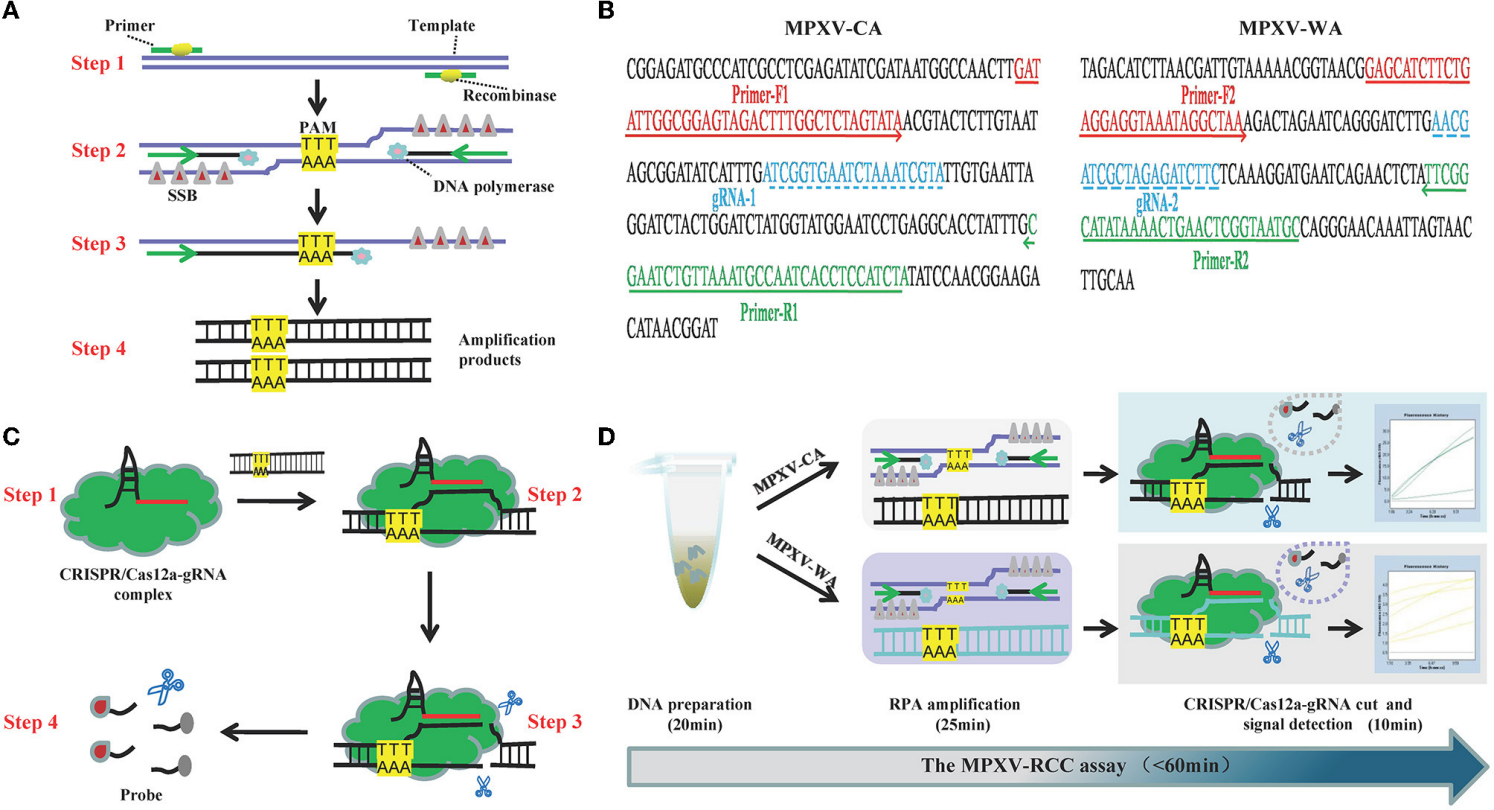
Schematic illustration of the MPXV-RCC technique. **(A)** Outline of RPA amplification for MPXV. Step 1: recombinase–primer complex specifically recognizes the target sequence containing a PAM site. Step 2: a D-loop is formed by the combination of SSB and DNA chain, and then, DNA polymerase activates the isothermal amplification. Step 3: displaced D-loop keeps the DNA open when the amplification continues. Step 4: products are generated exponentially. **(B)** Schematic illustration of CRISPR/Cas12a-gRNA detection. Step 1: Cas12a enzyme couples with gRNA to constitute CRISPR/Cas12a-gRNA complex. Step 2: gRNA sequence combines with target DNA near the PAM site. Step 3: Cas12a enzyme is activated to cut the template chain. Step 4: ssDNA is cleaved non-specifically by the Cas12a enzyme, and it could be available as a fluorescent probe. **(C)** Primer and gRNA sequence alignment on the MPXV segment. Nucleotide sequences of MPXV-CA and MPXV-WA are indicated. The locations of primers and gRNA are underlined, and the arrowheads show the amplified direction of primers. **(D)** Outline of MPXV-RCC assay workflow. MPXV-RCC diagnosis platform employs three steps: DNA preparation, RPA amplification, and CRISPR/Cas12a-gRNA cut and signal detection.

The details of RPA primers and gRNA are listed in Table 1 and Figure 1C. The specificity of two RPA primer sets was verified using NCBI BLAST analysis. Two gRNA strands for MPXV-CA and MPXV-WA were designed based on the MPXV-RCC principle. Furthermore, the probe used for fluorescence detection was labeled at the 5′ end with FAM fluorophore and at the 3′ end with a BHQ1 quencher. The oligonucleotides were synthesized by Sangon Biotechnology (Shanghai, China).

**Table 1:** Primers, gRNA, and probe of the MPXV-RCC assay.

**Pathogens**	**Objects**	**Sequences and modifications (5^′^-3^′^)**	**Length^b^**
MPXV-CA	Primer-F1	GATATTGGCGGAGTAGACTTTGGCTCTAGTATA	33 nt
	Primer-R1	TAGATGGAGGTGATTGGCATTTAACAGATTCG	32 nt
	gRNA-1	UAAUUUCUACUAAGUGUAGAUAUCGGUGAAUCUAAAUCGUA	41 mer
MPXV-WA	Primer-F2	GAGCATCTTCTGAGGAGGTAAATAGGCTAA	30 nt
	Primer-R2	GCATTACCGAGTTCAGTTTTATATGCCGAA	30 nt
	gRNA-2	UAAUUUCUACUAAGUGUAGAUAACGAUCGCUAGAGAUCUUC	41 mer
	Probe^a^	FAM-TATTAT-BHQ1	6 mer

^a^probe, 5′ and 3′ ends were labeled with FAM and BHQ1. The probe was shared in both clade detection. ^b^nt, nucleotide; mer, monomeric unit.

### The standard RPA reaction

The RPA pre-amplification process was carried out in a 25 μl reaction mixture according to the manual instruction. In brief, 13 μl of buffer I, 2.5 μl of enzyme mixture, 1.2 μl of forward primer (10 μM) and reverse primer (10 μM), 1.25 μl of buffer II, and 3.6 μl of distilled water were first added into a tube to construct the reaction system. Then 1 μl DNA template (1.0 × 10^3^ copies) and 1.25 μl reaction trigger were added to activate the reaction at 38°C for 25 min. Enterovirus (JACDC-1) ( 1.0 × 10^3^ copies/μl) and distilled water acted as negative and blank controls. The cross-reaction test of primers was carried out between MPXV-CA and MPXV-WA to verify the discernibility of this assay. The RPA amplicons were analyzed using electrophoresis on the 2% agarose gel. The RPA amplification was implemented at a constant temperature from 35 to 42°C with a 1°C increase to optimize the experimental condition.

### CRISPR/Cas12a-based assay

The CRISPR/Cas12a-based assay was carried out using Cas12a protein mixing with gRNA, which was implemented similarly to a previous study (14). The reaction system contained 0.5 μl of gRNA (10 μM), 0.5 μl of fluorescence reporter (5′-FAM-TATTAT-BHQ1-3′, 10 μM), 5 μl of reaction buffer (10 × ), 2 μl of RPA amplicon, 0.3 μl of Cas12a enzyme (20 μM), and 41.7 μl of distilled water to make a final volume of 50 μl. The released fluorescence signals were detected by a fluorescence detector.

### Sensitivity analysis of the MPXV-RCC assay

Recombination plasmids and pseudovirus were used to assess the limit of detection (LoD) of the MPXV-RCC assay. MPXV-CA and MPXV-WA recombination plasmids were serially diluted from 1.0 × 10^5^ to 1.0 × 10^2^ copies/μl with 10-fold intervals, and then, six dilutions with concentrations of 50 copies/μl, 25 copies/μl, 10 copies/μl, 5 copies/μl, 2.5 copies/μl, and 1 copy/μl were acquired successively. All the dilutions were tested in triplicate to validate their consistency with distilled water as the blank control. Similarly, serial dilutions of MPXV pseudovirus ranging from 1.0 × 10^5^ to 1 copy/μl were obtained and examined following the settlement process of the plasmid. Similarly, the LoD of MPXV-RCC in pseudovirus was also confirmed.

### MPXV-RCC detection in spiked blood specimens

The practicability of the MPXV-RCC assay was evaluated with spiked human blood samples. The spiked blood samples were prepared by inoculating the serially diluted pseudovirus solutions into blood samples. The DNA of the simulated specimen was extracted by the EasyPure Viral DNA/RNA kit (TransGen biotechnology, Beijing, China) and eluted in a 50 μl of buffer solution. Finally, 1 μl of obtained nucleic acid acted as a template for the MPXV-RCC assay. According to the conversion, the final concentration of pseudovirus in the spiked blood samples for MPXV-RCC detection was 1.0 × 10^5^ 1 copies/reaction. Distilled water and non-spiked blood samples were taken for quality control of the experiment. Three parallel trials were performed to explore the detection threshold of the MPXV-RCC diagnosis platform in artificial specimens.

### Specificity evaluation of the MPXV-RCC assay

The specificity of the MPXV-RCC assay was explored with non-MPXV pathogens and MPXV-positive specimens. Negative samples are displayed in Supplementary Table S1. Due to the lack of proper experimental resources, positive samples were simulated by blending MPXV pseudovirus and human blood. The concentration of MPXV pseudovirus used here was 1.0 × 10^2^ copies/μl, and the strains were singly mixed with blood samples collected from 10 human subjects. The operation procedures of the specimen mixing and genomic DNA extraction referred to that of the spiked sample. Two repeated tests were conducted to determine the accuracy of this method.

## Result

### Overview of the MPXV-RCC technique

The principle of the MPXV-RCC technique is illustrated in Figure 1. In brief, after the genomic DNA is added to the RPA reaction mixture, the target sequence will be amplified at 38°C according to operating instructions (Figure 1A). In the RPA method, the dissociation of dsDNA relies on the activity of recombinase instead of a denaturation step. Initially, the recombinase–primer complex is constituted by binding recombinase to primers, which then seeks homologous sequences of primers on the target dsDNA segment. Following the displaced DNA strand stabilized by the SSB (single-stranded binding proteins) which formed a D-loop, the recombinase disassociated with the primers and the DNA polymerase binds to the 3′ end of the primer to initiate the amplification of target sequences. Thus, numerous target DNA sequences will be produced within 25 min. Particularly, in this study, the target sequences contained a specific protospacer adjacent motif (PAM) site (TTT), which could be employed for the following CRISPR detection.

During the detection stage, first, the Cas12a protein integrates with gRNA to constitute a CRISPR/Cas12a-gRNA complex, which can recognize the specific sequence adjoining the PAM site under the guidance of gRNA (Figure 1B). The Cas12a is therewith activated to cleave the template chain and simultaneously trim the ssDNA molecule due to its non-specific trans-cleavage capacity, which results in the release of fluorescent signals. The entire detection process of the MPXV-RCC technique consists of three steps, including rapid DNA preparation (20 min), RPA amplification (25 min), and CRISPR-based detection (10 min) (Figure 1D), which can be completed within 60 min.

### Confirmation of the MPXV-RCC method

**Figure 2:**
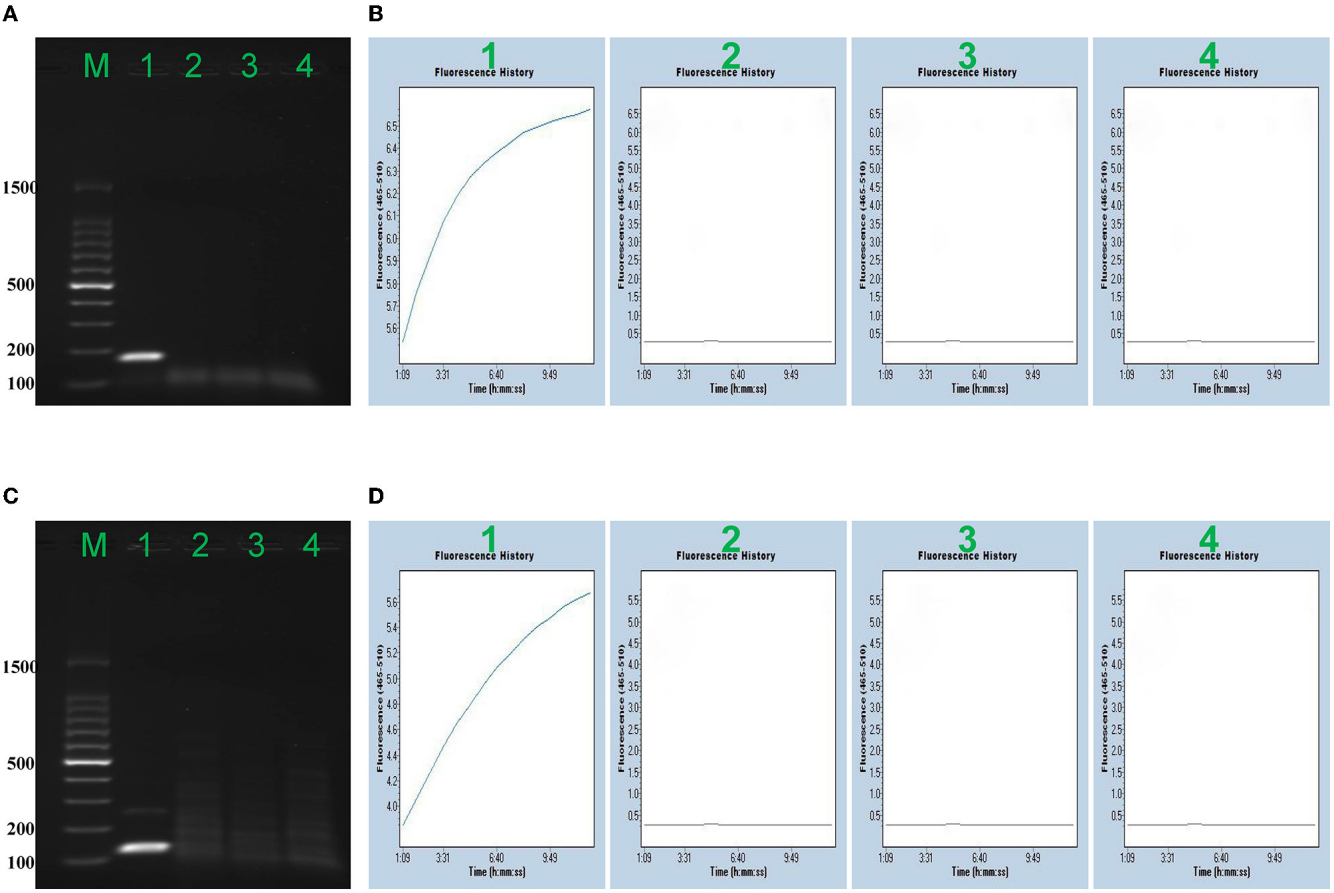
Confirmation of the MPXV-RCC assay. Agarose gel electrophoresis (AAG) indicated the RPA results, and fluorescence signal (FS) detection showed further CRISPR results. AAG **(A)** and FS **(B)** exhibited the detection of MPXV-CA. AAG **(A)**/FS **(B)** 1–4 represented MPXV-CA plasmid, MPXV-WA plasmid, enterovirus (JACDC-1), and distilled water, respectively. AAG **(C)** and FS **(D)** exhibited the detection of MPXV-WA. AAG **(C)**/FS **(D)** 1–4 singly represented MPXV-WA plasmid, MPXV-CA plasmid, enterovirus (JACDC-1), and distilled water.

To verify the reliability of the MPXV-RCC method, the RPA primers of MPXV-CA and MPXV-WA were screened based on the images of the specific target band on 2% agarose gel, and each gRNA for CRISPR/Cas12a-based detection was validated according to the fluorescence intensity monitored by a fluorescence detector. As shown in Figure 2, the primers and gRNA of both clades of MPXV showed excellent performance in testing each target. The strong brightness and fluorescence signals could only be observed in the reactions loaded with positive plasmids DNA, while other reactions spiked with non-MPXV templates (enterovirus and distilled water) did not show the corresponding experimental characteristics. Furthermore, each primer set specially amplified its target template, and no cross-reaction was observed. Thus, the primer sets and gRNAs were suitable candidates for the development of MPXV-RCC assay to diagnose MPXV infection.

### Optimal temperature for the MPXV-RCC method

To determine the optimal reaction temperature of MPXV-RCC at the amplification step, MPXV-CA- and MPXV-WA-RPA reactions were performed at temperatures from 35 to 42°C (interval of 1°C) using MPXV-CA and MPXV-WA recombination plasmids (1.0 × 10^2^ copies/μl), respectively. The results from electrophoresis (Supplementary Figure S1) illustrated that 37 to 40°C were the better options for the MPXV-CA-RPA reaction and 37 to 39°C for MPXV-WA. As a result, a temperature of 38°C was selected to implement the MPXV-RCC method at the isothermal reaction stage.

### Sensitivity estimation of the MPXV-RCC assay

**Figure 3:**
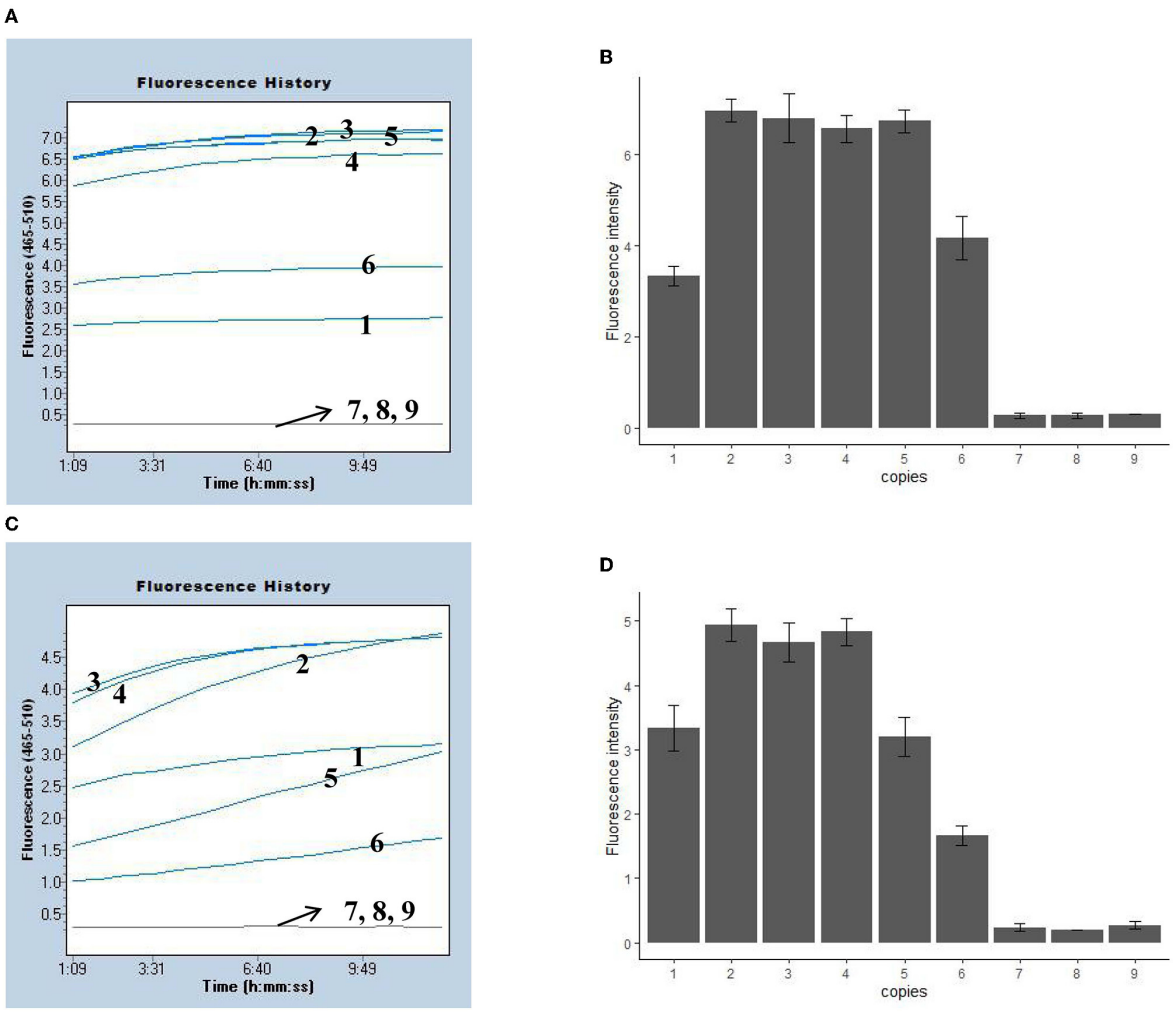
Sensitivity of the MPXV-RCC assay in recombinant plasmids. **(A, C)** Indicate the fluorescence results of the MPXV-RCC assay for MPXV-CA and MPXV-WA detection with recombinant plasmids, respectively. **(B, D)** Show the statistical results of fluorescence intensity for two clades. Lines/bars 1–9 represent the results with plasmid levels of 1.0 × 10^3^, 1.0 × 10^2^, 50, 25, 10, 5, 2.5, 1 copies/reaction, and blank control, respectively.

We evaluated the LoD of the MPXV-RCC assay using recombination plasmids and pseudovirus, and various dilutions of MPXV-CA and MPXV-WA plasmids (ranging from 1.0 × 10^5^ 1 copies per reaction) were tested. Using the plasmid as a template, the LoD of the MPXV-RCC assay was down to five copies per reaction for both MPXV-CA and MPXV-WA detection (Figure 3). These tests were performed with three duplicates. Using the DNA templates extracted from pseudovirus, the MPXV-RCC assay could detect down to five copies of pseudovirus for MPXV-CA but 10 copies for MPXV-WA (Supplementary Figure S2).

### Validation of the MPXV-RCC detection system using MPXV-spiked clinical samples

To examine the feasibility of the MPXV-RCC assay in clinical settings, the spiked human blood samples with MPXV pseudovirus were applied due to the unavailability of clinically positive patients. Similar to the pure templates, the LoD of the MPXV-RCC assay for CA and WA clades of MPXV were 5 and 10 copies per reaction in spiked blood samples, respectively, as well (Supplementary Figure S3), while the lower concentrations, non-spiked sample, and distilled water gave negative results.

### Specificity of the MPXV-RCC method

**Figure 4:**
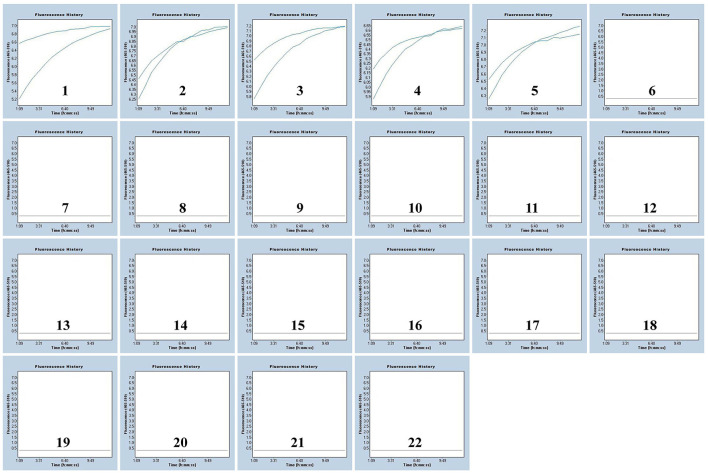
Specificity of the MPXV-RCC assay for MPXV-CA detection. The results of two repeated tests are shown in the figure. Graphs 1–5: MPXV-CA agents (spiked clinical samples). Graphs 6–21: influenza A virus, enterovirus, adenovirus, coronavirus, dengue virus, Epstein-Barr virus, hepatitis B virus, human rhinovirus, herpes simplex virus-1, influenza B virus, measles virus, parainfluenza virus, rubella virus, respiratory syncytial virus, Visna virus, and vesicular stomatitis virus, separately. Graph 22, blank control.

To further determine the specificity of our assay, we extracted DNA templates from spiked MPXV pseudovirus samples and various non-MPXV strains. Positive signals were only observed in MPXV-spiked samples but not in the non-MPXV templates, suggesting their accuracy for both clades of MPXV detection (Figure 4; Supplementary Figure S4). These results suggested that the assay developed here could specifically detect MPXV.

## Discussion

The outbreak of monkeypox has caused another public health emergency during the COVID-19 pandemic. To control the further spread of this disease, simple and efficient methods for MPXV detection are increasingly in demand. In this study, a rapid, accurate, and sensitive MPXV diagnosis platform coupling RPA amplification with CRISPR/Cas12a-gRNA detection has been devised and named MPXV-RCC. In the MPXV-RCC system, the target sequences were pre-amplified by RPA assay at a constant temperature. Then, the trans-cleavage characteristic of the Cas12a protein is activated to cut the RPA products and digest the detection probe in the mixtures. Finally, the diagnostic result could be judged by observing fluorescence intensity.

In this study, the reaction conditions of the MPXV-RCC assay, including RPA reaction and CRISPR/Cas12a-gRNA detection, were optimized. The isothermal RPA reaction only needed a simple heating instrument, such as a water bath, to maintain the reaction at a constant temperature (38°C), and the MPXV products could be amplified exponentially within 25 min. CRISPR/Cas12a-gRNA detection was carried out at 37°C for 10 min by employing a real-time fluorescence instrument, and the procedure of detection was simple and rapid. The whole process of the MPXV-RCC assay, including DNA preparation (20 min), RPA isothermal amplification (25 min), and CRISPR/Cas12a-gRNA detection (10 min), could be performed within 1 h. Therefore, the established method could be applied as a rapid tool for the diagnosis of MPXV infection, which might be especially suitable for the point-of-care (POC) test.

The recombination plasmids and pseudovirus were used to evaluate the sensitivity of the MPXV-RCC method, and an excellent result was obtained. This assay could detect plasmid templates as low as 5–10 copies/reaction when identified for MPXV-CA and MPXV-WA, which is >100 times more sensitive than the conventional PCR method and has a better sensitivity than the real-time PCR assay (20, 21). Particularly, PCR-based assays need more reaction time. In addition, due to no available clinically positive MPXV-infected patients in our country, the clinical feasibility of the MPXV-RCC assay was further validated using spiked samples. This technique was able to detect < 10 copies of pseudovirus in simulated blood specimens, and it demonstrated that the established diagnostic platform was supersensitive in clinical settings. In addition to the low LoD, the novel-designed assay appeared specific. The identification ability of nucleic acid extracted from MPXV-positive samples and negative species could explain the specificity of the MPXV-RCC method. Particularly, the specificity was guaranteed by combining gRNA with primers, which recognized the MPXV sequence precisely. The results displayed that the MPXV-RCC assay could differentiate MPXV-CA and MPXV-WA and had no cross-reactivity with other pathogenic microorganisms. Hence, the diagnostic method relied on CRISPR/Cas12a-gRNA detection exhibited excellent accuracy.

Some isothermal amplification methods including LAMP and RPA have been exploited to monitor MPXV for the past few years (21, 22). Compared with LAMP, the MPXV-RCC method possesses a slightly higher sensitivity. Primer design and screening of this assay are easier than that of LAMP, which needs three pairs of primers (21). Similarly, the MPXV-RCC platform reveals a preferable sensitivity in comparison with the RPA-only detection (23), and a previous study also indicated that the CRISPR/Cas12a-gRNA detection could enhance the sensitivity of the diagnosis assay (24). In addition, non-specific amplification is an inherent defect of RPA reaction, whereas the CRISPR/Cas12a-gRNA detection can solve this problem and make the method more specific. For result judgment, fluorescence signal detection used in the MPXV-RCC method is more exact than the colorimetric indicator, which was usually used in isothermal amplification tests. Those tests obtain the result based on color change, and it will face trouble when the content of the target sequence is very low (25, 26).

In summary, we chose the target sequence elaborately, devised the primers and gRNA to establish a reliable CRISPR/Cas12a-based method for the diagnosis of MPXV infection, and subsequently verified the practicability of the proposed assay in this study. The MPXV-RCC diagnosis platform developed here shows merits in simplicity, rapidity, specificity, and sensitivity, which could detect 5–10 copies of DNA per reaction in recombination plasmids, pseudovirus, and spiked clinical samples, and the detection time only needs 60 min. Thus, the MPXV-RCC assay provides a useful diagnostic tool for the timely diagnosis of monkeypox cases in clinical settings.

## Data availability statement

The original contributions presented in the study are included in the article/Supplementary material, further inquiries can be directed to the corresponding authors.

## Ethics statement

The study involving the samples of human origin was approved by the Institutional Review Board of Wuhan Center for Disease Control and Prevention (WHCDCIRB-K-2021038). The blood specimens were extracted from 10 healthy people who had consented the experiments and signed the ICF (Informed Consent Form).

## Author contributions

LG, XL, JL, and YiW devised the experimental scheme. LG, XC, and YiW wrote and reviewed the manuscript. LG performed the experiments. YimW and YiW collected partial samples and reagents. LG and XL analyzed the experimental data. All authors contributed to the article and approved the submitted version.
