# Supplementary material for: Rapid, sensitive, and highly specific detection of monkeypox virus by CRISPR-based diagnostic platform
Source: Front Public Health. 2023 Jun 27;11:1137968. doi: 10.3389/fpubh.2023.1137968 (PMC10335395; doi:10.3389/fpubh.2023.1137968)
Supplement: Supplementary file 1 [file Data_Sheet_1.doc]

Supplementary Material

**Rapid, Sensitive and Highly Specific Detection of Monkeypox Virus by CRISPR-based Diagnostic Platform**

**Lin Gong^1^**^†^**, Xiaomin Chen****^1^**^†^**, Yimei Wang^1^, Jiansheng Liang^1^, Xiaoli Liu^1^*, Yi Wang^2^***

^1^Department of Disinfection and Pest Control, Wuhan Center for Disease Control & Prevention, Wuhan, Hubei, China. ^2^Experimental Research Center, Capital Institute of Pediatrics, Beijing, China.

***Correspondence:**

Xiaoli Liu

E-mail: liuxiaoli20851@126.com.

Yi Wang (handing the correspondence)

E-mail: wildwolf0101@163.com.

**1. Supplementary Tables**

**Table S**1. Pathogens used in this study.

| Pathogens | Forms | Sources# | No. of isolates |
| --- | --- | --- | --- |
| MPXV-CA | recombination plasmid | TH | 1 |
| MPXV-WA | recombination plasmid | TH | 1 |
| MPXV | pseudovirus | SB | 1 |
| influenza A virus | nucleic acid | JACDC | 10 |
| influenza A virus | nucleic acid | CIP | 1 |
| enterovirus | nucleic acid | JACDC | 10 |
| adenovirus | nucleic acid | CIP | 1 |
| coronavirus | nucleic acid | CIP | 1 |
| dengue virus | nucleic acid | CIP | 1 |
| epstein-barr virus | nucleic acid | CIP | 1 |
| hepatitis B virus | nucleic acid | CIP | 1 |
| human rhinovirus | nucleic acid | CIP | 1 |
| herpes simplex virus-1 | nucleic acid | CIP | 1 |
| influenza B virus | nucleic acid | CIP | 1 |
| measles virus | nucleic acid | CIP | 1 |
| parainfluenza virus | nucleic acid | CIP | 1 |
| rubella virus | nucleic acid | CIP | 1 |
| respiratory syncytial virus | nucleic acid | CIP | 1 |
| visna virus | nucleic acid | CIP | 1 |
| vesicular stomatitis virus | nucleic acid | CIP | 1 |

^#^TH, Tianyi-Huiyuan Biotechnology (Beijing) Co., Ltd. SB, Sangon Biotechnology (Shanghai) Co., Ltd. JACDC, Ji’an Municipal Center for Disease Control and Prevention. CIP, Capital Institute of Pediatrics, Beijing.

## 2. Supplementary Figures


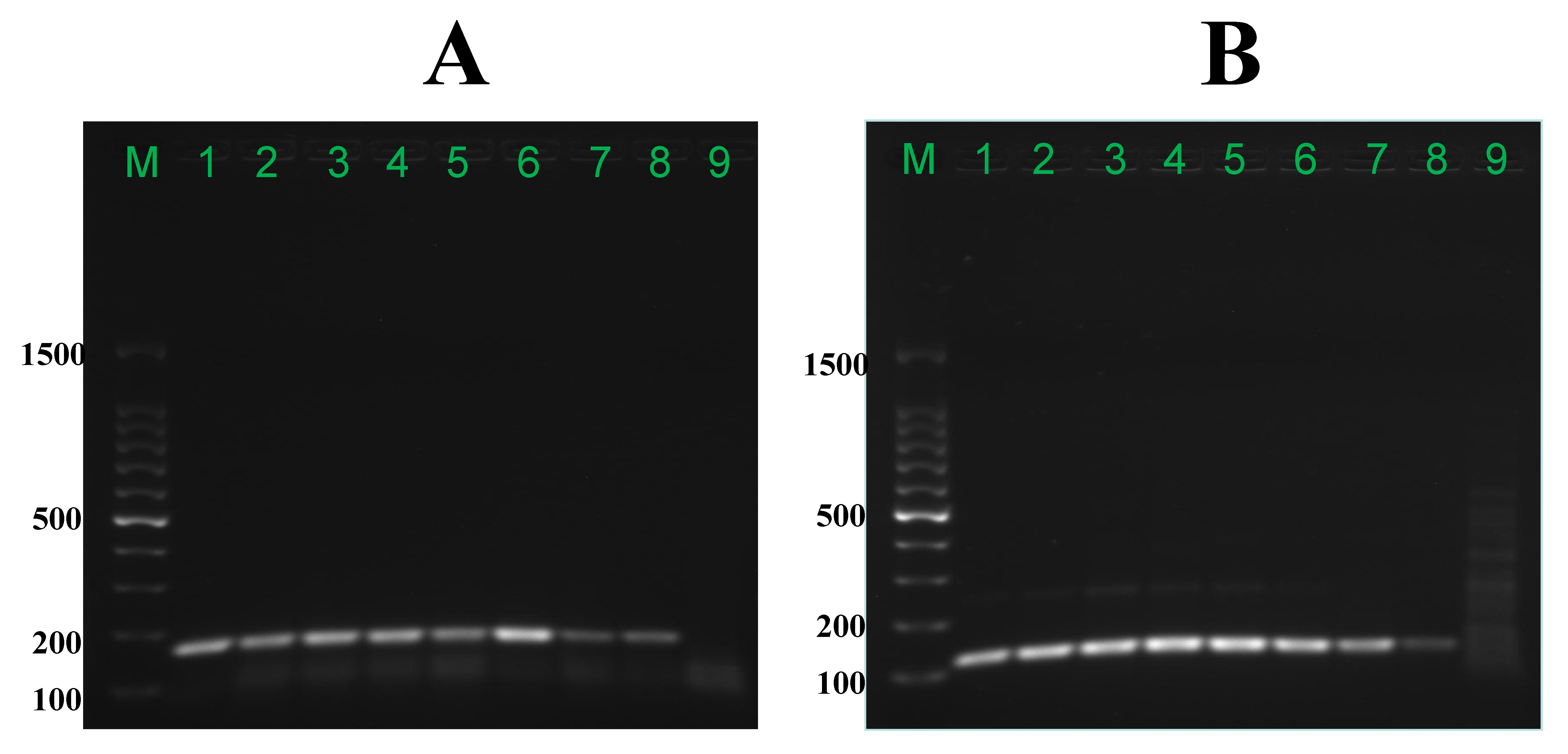


**Figure S1. The optimum selection of temperature for RPA reaction.** The RPA reaction results of MPXV-CA (A) and MPXV-WA (B) at different temperatures were showed by AAG method and AAG Lanes 1-8 represented the results at temperature from 35 to 42℃ with 1˚C increments, respectively. Lane 9, distilled water. M, 100 bp marker.


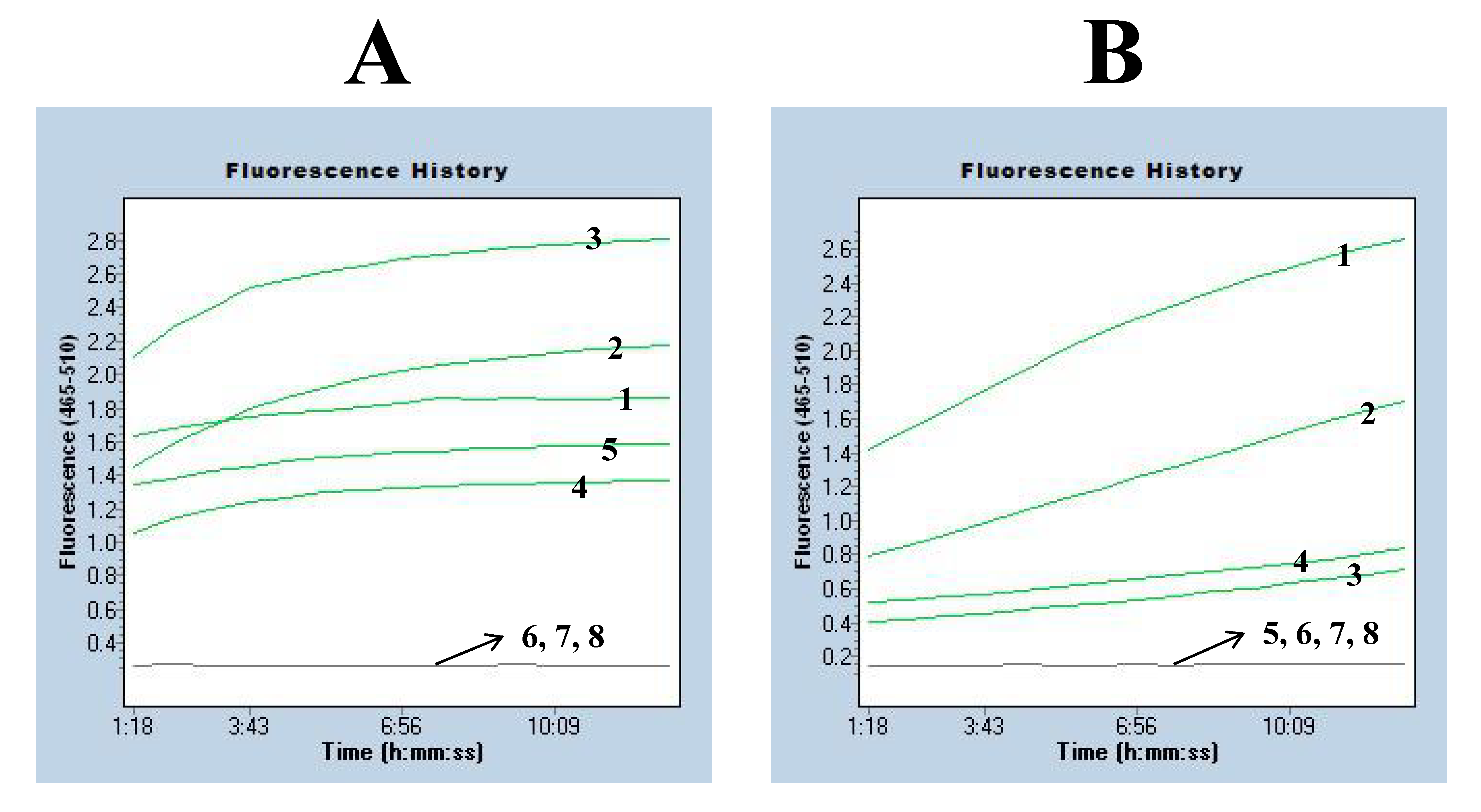


**Figure S2. Threshold of MPXV-RCC assay with** [**pseudovirus**](javascript:;) **templates.** Figures (A) and (B) respectively denoted the application of MPXV-RCC for MPXV-CA and MPXV-WA with pseudovirus templates, respectively. Signals 1～8 represented results of pseudovirus templates with DNA concentrations of 1.0×10^2^, 50, 25, 10, 5, 2.5, 1 copies/ reaction, and blank control, respectively.


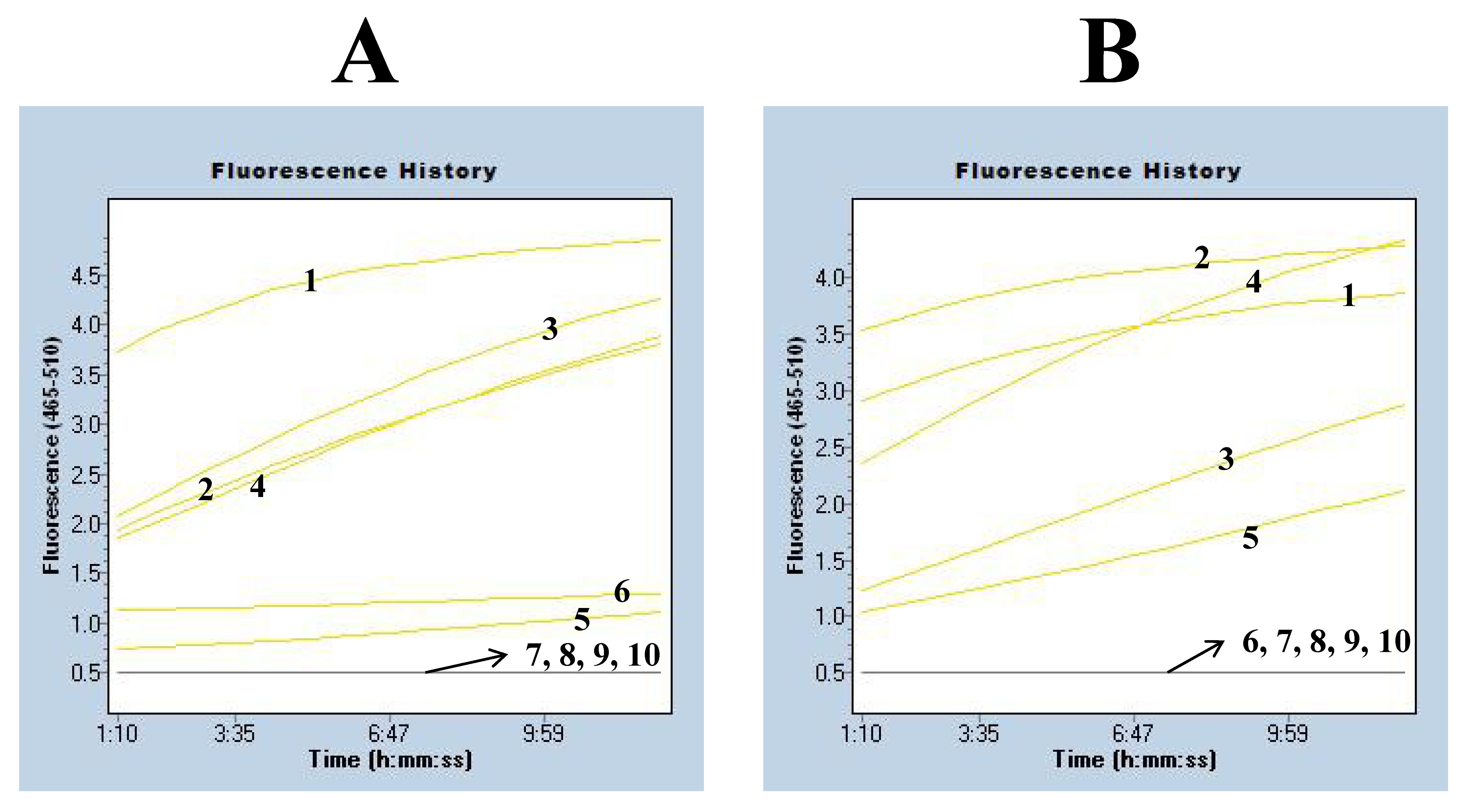


**Figure S3. LoD of MPXV-RCC assay in spiked blood samples.** Figures (A) and (B) meant results of the MPXV-RCC assay for MPXV-CA and MPXV-WA diagnosis in spiked blood samples, respectively. Signals 1～10 showed spiked samples DNA levels of 1.0×10^3^, 1.0×10^2^, 50, 25, 10, 5, 2.5, 1 copies/ reaction, non-spiked sample and blank control, singly.

**
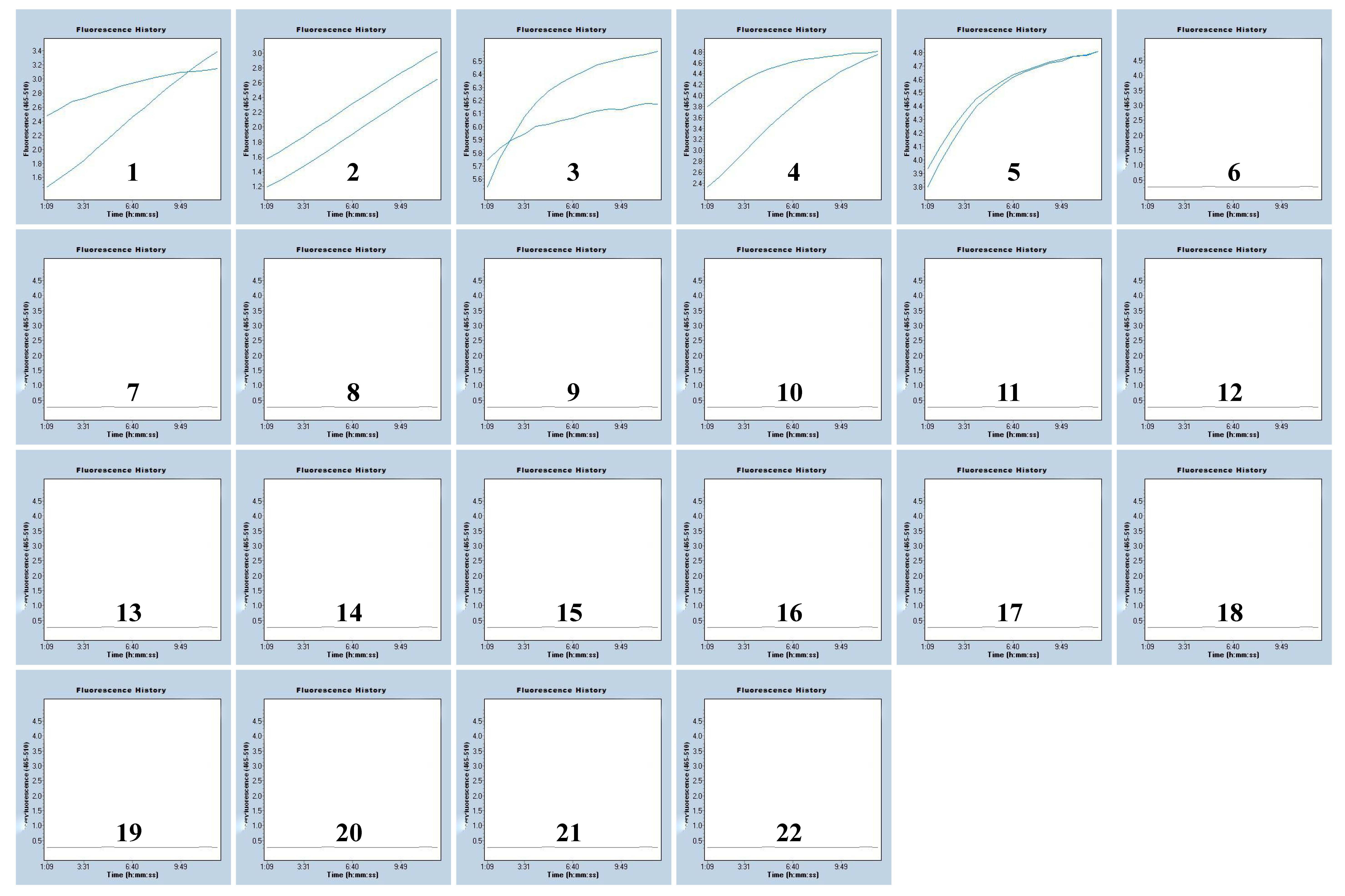
**

**Figure S4. Specificity of MPXV-RCC assay for MPXV-WA detection**. The results of two repeated tests were showed in the figure. Graphs 1-5, MPXV-WA agents (spiked clinical samples). Graphs 6-21, influenza A virus, enterovirus, adenovirus, coronavirus, dengue virus, epstein-barr virus, hepatitis B virus, human rhinovirus, herpes simplex virus-1, influenza B virus, measles virus, parainfluenza virus, rubella virus, respiratory syncytial virus, visna virus, vesicular stomatitis virus, separately. Graph 22, blank control.
